# The Impact of the COVID-19 Pandemic on Incidences of Atrial Fibrillation and Electrical Cardioversion at a Tertiary Care Emergency Department: An Inter- and Intra-year Analysis

**DOI:** 10.3389/fmed.2020.595881

**Published:** 2020-12-04

**Authors:** Sebastian Schnaubelt, Hans Domanovits, Jan Niederdoeckl, Nikola Schuetz, Filippo Cacioppo, Julia Oppenauer, Alexander O. Spiel, Anton N. Laggner

**Affiliations:** ^1^Department of Emergency Medicine, Medical University of Vienna, Vienna, Austria; ^2^Vienna Health Care Group, Department of Emergency Medicine, Clinic Ottakring, Vienna, Austria

**Keywords:** atrial fibrillation, electrical cardioversion, COVID-19, lockdown, critical care

## Abstract

**Background:** National authorities have introduced measures as lockdowns against spreading of COVID-19 and documented incidences of multiple non-COVID-19 diseases have dropped. Yet, data on workload dynamics concerning atrial fibrillation and electrical cardioversion whilst a national lockdown are scarce and may assist in future planning.

**Methods:** Documented cases of atrial fibrillation and respective electrical cardioversion episodes at the Emergency Department of the Medical University of Vienna, Austria, from 01/01/2020 to 31/05/2020 were assessed. As reference groups, those incidences were calculated for the years 2017, 2018, and 2019. Inter- and intra-year analyses were conducted through Chi-square test and Poisson regression.

**Results:** A total of 2,310 atrial fibrillation-, and 511 electrical cardioversion episodes were included. We found no significant incidence differences in inter-year analyses of the time periods from January to May, or of the weeks pre- and post the national lockdown due to the COVID-19 pandemic. However, the intra-year analysis of the year 2020 showed a trend toward decreased atrial fibrillation incidences (rate-ratio 0.982, CI 0.964–1.001, *p* = 0.060), and significantly increased electrical cardioversion incidences in the post-lockdown period (rate ratio 1.051, CI 1.008–10.96, *p* = 0.020).

**Conclusion:** The decreased atrial fibrillation incidences are in line with international data. However, an increased demand of electrical cardioversions during the lockdown period was observed. A higher threshold to seek medical attention may produce a selected group with potentially more severe clinical courses. In addition, lifestyle modifications during isolation and a higher stress level may promote atrial fibrillation episodes to be refractory to other therapeutic approaches than electrical cardioversion.

## Introduction

During the ongoing corona virus disease 2019 (COVID-19) pandemic, national authorities have introduced measures to control the outbreak and avoid overloading already-strained healthcare resources such as intensive care units (1–4). Following recommendations by the World Health Organization (5), the response in Austria was local distancing, quarantines and a general public lockdown, starting in mid-March 2020. These precautionary measures have been extensively communicated through media, with information reaching a high percentage of the population. People subsequently isolated themselves, and the national healthcare focus was lying on outbreak control and treatment of infected patients. Therefore, it was hypothesized that non-COVID-19 diseases received less attention than usually. On the one hand, patients had to endure longer waiting times until definitive medical care, and on the other hand patients might have not been treated at all because of staying isolated, fearful of contracting COVID-19 in health care facilities (6).

International data indeed show decreased incidences and delayed treatment timeframes of acute coronary syndrome, stroke, or pulmonary embolism (2, 3, 7–10), but also an increase in out-of-hospital cardiac arrest (11), further stressing the hypothesis of patients not seeking medical attention too late or not at all (7).

The suspicion of dysrhythmia such as atrial fibrillation (AF) being associated with COVID-19 has been claimed before (12–14), and general AF incidences have been reported to have decreased during lockdown periods (1, 15). Since AF poses a prominent cardiovascular issue requiring medical attention and correct timely treatment (16), consequences of a pandemic and a subsequent lockdown on AF incidences, patient flow, and treatment details seem inevitable.

We therefore aimed at validating the reports from Holt et al. (1) in a Viennese tertiary care Emergency Department (ED), assessing the dynamics in the ED workload concerning AF, and clarifying if the need for interventions such as electrical cardioversion (eCV) follows the hypothesized decreased AF incidences.

## Methods

### Study Population and Data Acquisition

Within the present population-based retrospective observational study, primarily all cases of AF documented at the Emergency Department of the Medical University of Vienna, Austria, between 01/01/2020 and 31/05/2020 were included. As reference groups, all AF cases of the time periods of 01/01 to 31/05 of the years 2017, 2018, and 2019 were assessed. The respective data (incl. patients' age and gender) were culled from the department's anonymized case records, and AF was defined in accordance with the guidelines of the European Society of Cardiology (16). Apart from an age <18 years and a positive COVID-19 status, no specific exclusion criteria were applied. The Emergency Department of the Medical University of Vienna, Austria, was a designated non-COVID-19 hospital during the observational period. Due to fully anonymized data and because no direct clinical data were processed, informed consent was waived. The study was conducted under a positive ethics vote by the Ethics Committee of the Medical University of Vienna (EC-No. 1568/2014, registered at NCT03272620), and complies with the declaration of Helsinki.

### COVID-19 Dynamics

COVID-19 was first detected in Austria on 25/02/2020, and reached the city of Vienna on 27/02/2020. Details and dynamics of epidemiological data concerning the pandemic within the observation period (starting with the first case on 25/02/2020 until 31/05/2020) were obtained from official government records (4).

### Statistical Analysis

Incidences of AF and electrical cardioversion were calculated per week. Continuous data are presented as median and the respective interquartile range (IQR) and compared among subgroups using Mann-Whitney-*U*-test. Categorical parameters are presented as counts and percentages and were analyzed using Chi-square test including testing for linear association. For intra-year analyses of incidences in 2020, Poisson regression was conducted. Statistical significance was assumed through two-sided *p* < 0.05. Statistical analyses were performed using SPSS 24.0 (IBM SPSS, USA).

## Results

In the observational periods of January to May 2017, 2018, 2019, and 2020, a total of 2310 AF episodes were noted, of which 511 episodes (22.1%) necessitated electrical cardioversion. This leads to an average of 116 AF episodes and 26 electrical cardioversions per month. No significant differences were seen when comparing patients' age and gender in the single year periods of 2017, 2018, 2019, and 2020, and the cumulative values of 2017–2019 (Table 1).

**Table 1:** Patients' age and gender characteristics for the atrial fibrillation (AF) and electrical cardioversion episodes of the respective weeks 1–22 of the years 2017, 2018, 2019, 2020, and the cumulative values for 2017–2019 for comparison.

**AF episodes**	**2017**	**2018**	**2019**	**2017–2019**	**2020**	***p*-value**
**Age, years (IQR)**						
Weeks 1–22	75 (65–81)	74 (65–81)	75 (66–82)	75 (66–82)	76 (67–83)	0.354
Weeks 1–11	74 (64–79)	76 (65–80)	75 (65–83)	75 (65–81)	76 (66–82)	0.299
Weeks 12–22	75 (65–80)	75 (66–81)	75 (66–83)	75 (65–82)	76 (66–82)	0.301
**Male gender**, ***n*** **(%)**						
Weeks 1–22	288 (48.3)	281 (49.1)	307 (50.0)	876 (49.2)	261 (49.3)	0.293
Weeks 1–11	168 (50.3)	149 (49.0)	149 (48.9)	462 (49.0)	143 (49.3)	0.098
Weeks 12–22	126 (48.1)	133 (49.8)	154 (49.8)	419 (49.9)	117 (49.0)	0.109
**Electrical cardioversions**	**2017**	**2018**	**2019**	**2017–2019**	**2020**	***p*****-value**
**Age, years (IQR)**						
Weeks 1–22	67 (56–75)	69 (55–75)	65 (55–75)	67 (55–75)	63 (54–75)	0.180
Weeks 1–11	67 (56–77)	68 (54–74)	65 (55–74)	67 (55–76)	64 (55–77)	0.200
Weeks 12–22	66 (56–74)	69 (55–74)	64 (54–74)	66 (54–74)	63 (54–75)	0.198
**Male gender**, ***n*** **(%)**						
Weeks 1–22	76 (58.5)	74 (55.6)	83 (69.2)	233 (60.8)	77 (60.2)	0.092
Weeks 1–11	42 (59.2)	37 (56.1)	38 (69.1)	117 (60.9)	34 (60.7)	0.103
Weeks 12–22	34 (57.6)	37 (55.2)	45 (69.2)	116 (60.7)	43 (59.7)	0.099

*Continuous data are presented as median and the respective interquartile range (IQR) and compared among subgroups using Mann–Whitney–U–test*.

### Four-Year Dynamics

In an inter-year analysis of the observational periods within the years 2017, 2018, 2019, and 2020, only a trend, but no significant differences were found in AF incidences (596 vs. 571 vs. 614 vs. 529 episodes, respectively, *p* = 0.232). Thereby, similar results could be shown for the therapeutic approach of eCV (130 vs. 133 vs. 120 vs. 128 episodes, respectively, *p* = 0.479). Moreover, these results remained non-significant in a differentiated analysis of the weeks before- (weeks 1–11) and after (weeks 12–22) the national lockdown due to COVID-19 in 2020 (Table 2).

**Table 2:** The number of atrial fibrillation (AF) episodes and electrical cardioversions in an inter–year analysis.

**Number of AF episodes**	**2017**	**2018**	**2019**	**2020**	***p*-value**
Weeks 1–22	596	571	614	529	0.232
Weeks 1–11	334	304	305	290	0.174
Weeks 12–22	262	267	309	239	0.369
**Number of electrical cardioversions**	**2017**	**2018**	**2019**	**2020**	***p*****-value**
Weeks 1–22	130	133	120	128	0.479
Weeks 1–11	71	66	55	56	0.878
Weeks 12–22	59	67	65	72	0.457

*Weeks 12–22 of 2020 depict the COVID−19 pandemic period after the national lockdown. Categorical parameters are presented as counts and percentages and analyzed using Chi–square test. Statistical significance was defined by two-sided p < 0.05*.

### An Intra-Year Analysis of 2020

**Figure 1:**
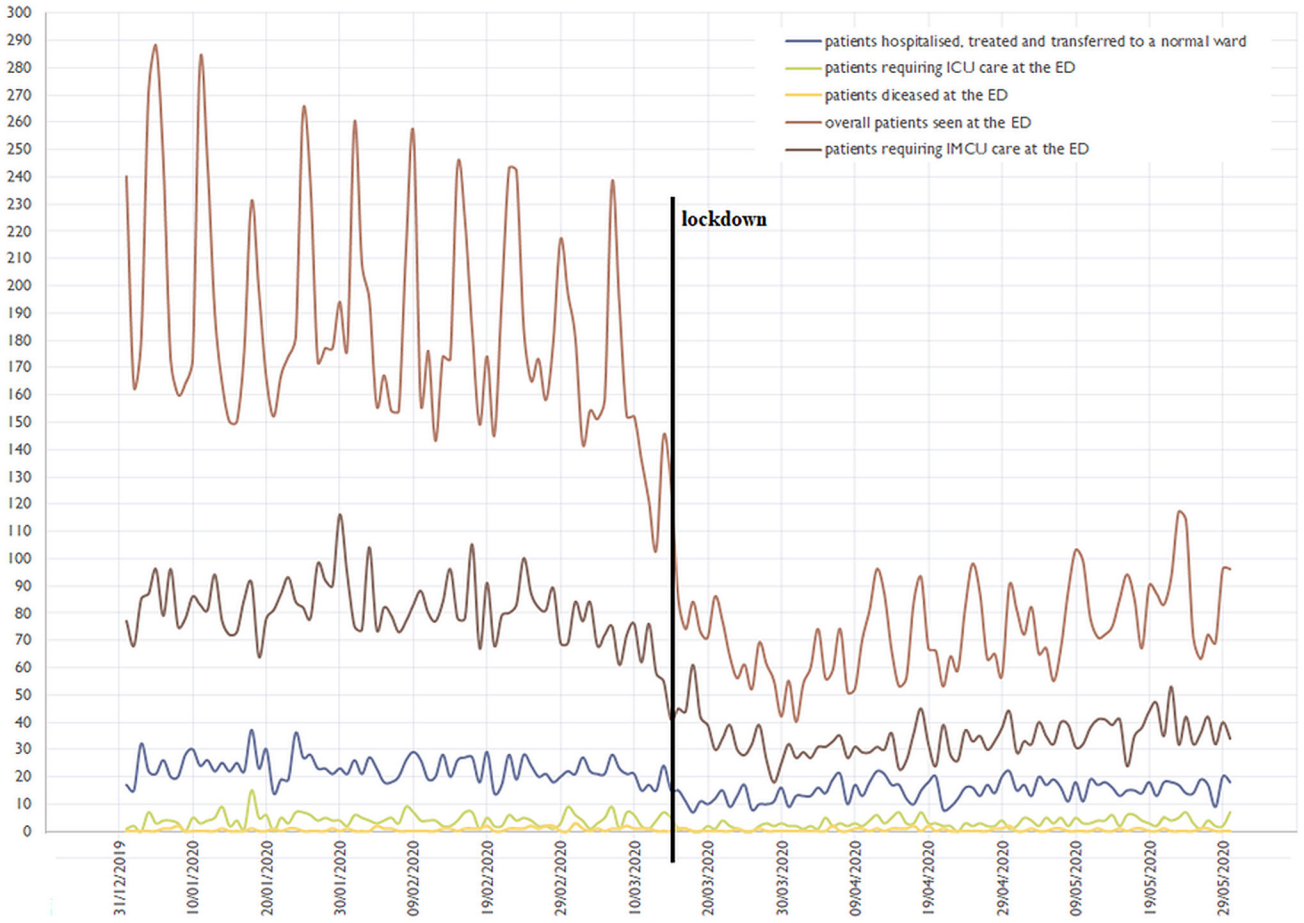
Depiction of the Emergency Department's (ED) patient flow from January to May 2020. The bold vertical line shows the national lockdown due to COVID-19 in mid-March; a decrease in overall- and intermediate care unit (IMCU) patients is seen. ICU, intensive care unit.

Within the weeks of January to May 2020, a total of 6,678 patients had been treated at the study center's ED for internal medicine diseases, resulting in an average of 1,336 patients per month or 44 patients a day, with a fluctuation toward higher numbers on weekends and national holidays, and lower numbers on work days. The number of ambulatory patients having been treated in an out-patient manner amounted to 4,897 (73.3%; 979 per month or 32 per day), that of admitted patients to 1,779 (26.6%; 355 per month or 12 per day). A more detailed analysis revealed a noticeable damper in overall patient numbers and numbers of patients requiring intermediate care unit (IMCU) treatment with the introduction of the national lockdown in mid-March 2020 (Figure 1).

**Figure 2:**
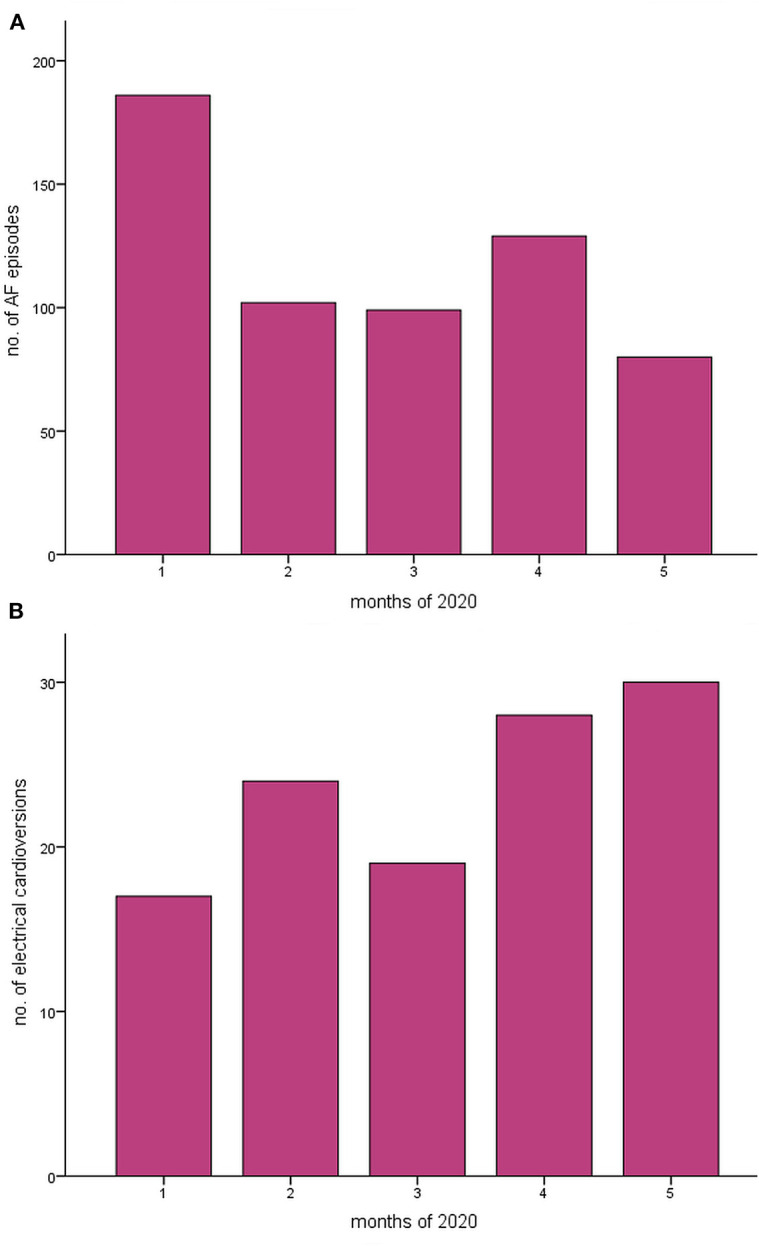
The incidences of **(A)** atrial fibrillation (AF) and **(B)** of electrical cardioversion in an intra-year analysis of the year 2020 National lockdown due to COVID-19 was conducted in mid-March. Poisson regression analysis was used to compare weeks 1–22 of 2020. A trend toward a decrease in AF could be found, corresponding to a rate ratio of 0.982 [95% CI 0.964–1.001, *p* = 0.060], and a significant increase in electrical cardioversions was seen, corresponding to a rate ratio of 1.051 [95% CI 1.008–1.096, *p* = 0.020].

**Figure 3:**
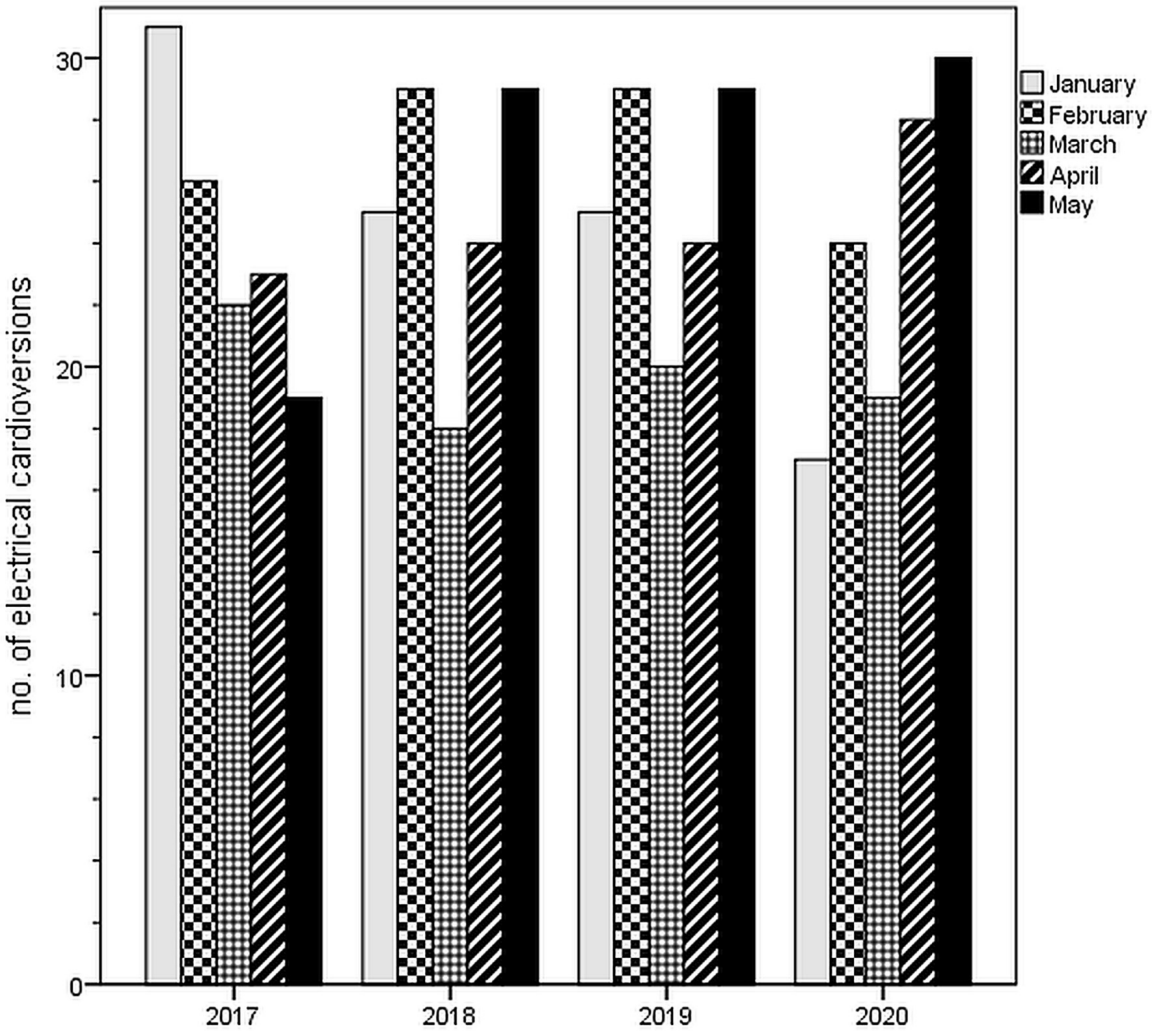
Numbers of electrical cardioversions from January to May in the years 2017, 2018, 2019, and 2020 (corresponding to Table 1). For a detailed analysis of the year 2020, see Figure 2.

Poisson regression analysis for the weeks stratified in before- and after the national lockdown revealed a trend toward a decrease in the incidence of AF (rate ratio 0.982, CI 0.964–1.001, *p* = 0.060) (Figure 2). Of importance, the same analysis conducted for eCV episodes yielded a significant increase toward the lockdown period (rate ratio 1.051, CI 1.008–10.96, *p* = 0.020) (Figure 3); this continuous rise in eCV numbers is unique to the year of 2020.

## Discussion

The presented data highlight the notch in general patient flow in our study center during the lockdown due to the COVID-19 pandemic. Moreover, we demonstrate a decrease of AF-, but an increase in eCV incidences from the pre-lockdown- toward the lockdown phase within the year 2020. To our knowledge, this analysis presents the first data on AF dynamics in a tertiary non-COVID-19 ED during the COVID-19 pandemic.

### A Decreased Workload of Specialized Centers

Our data on a designated non-COVID-19 tertiary care university hospital ED that serves—amongst others—as a primary contact point for acute coronary syndrome, stroke and as a cardiac arrest center in the city of Vienna, showed a decreased general patient flow and an associated reduced workload, being in accordance with international literature: Numbers of patients with the described diseases have dropped during national lockdown measures in numerous countries, including Austria. A fear of COVID-19 infection and a “watchful waiting” approach of medical caregivers toward less-symptomatic patients have been suggested as major drivers for this (2, 3, 7–10), also acting as a reasonable explanation for our findings. This effect might be gravest in ambulatory out-patient-department patients, not seeking medical attention themselves anymore (Figure 1), bearing the risk of a high rate of undetected diseases actually requiring treatment. To countersteer, a public information campaign addressing this dilemma in times of lockdowns might be reasonable. Of utmost importance, whenever COVID-19 numbers should rise to a state that tertiary care centers cannot longer be reserved as “non-COVID-19” facilities, the dilemma might become even worse: General COVID-19 patients and especially those in need of tertiary care (e.g., extra-corporal membrane oxygenation) could further crowd out others in need.

### Dropping AF Rates Fitting in the Picture

Before the background of AF as a possible complication of COVID-19 (12), it seems imperative not to oversee non-COVID-19 AF patients within the bigger picture. Even though our trend of decreased non-COVID-19 AF rates in an inter-year analysis did not reach statistical significance, our findings are in line with recent international data: Holt et al. report on a nationwide 47%-decrease in new-onset AF coded by the registry-based healthcare system, and warn of complications of underdiagnosis (1). Again, fear of contagion and a higher threshold of seeking medical attention has been discussed as the main reason (1, 15). We strengthen these data with our intra-year analysis of 2020 showing a trend toward a drop of AF incidences in the lockdown period (rate ratio 0.982, CI 0.964–1.001, *p* = 0.060).

Our data on decreasing AF rates not reaching statistical significance might have various explanations:

First, various factors may counteract the effect of the general reluctance of presenting to an ED or calling emergency medical services: Lifestyle modifications such as higher calories intake, a lack of physical activity or higher stress levels have all been reported both as being highly prevalent in lockdown periods, and as AF facilitators (1, 17–19). Indeed, severe emotional stress has been noted in populations after COVID-19 lockdowns (20, 21), and mental health seems to be affected (22). In particular, economic insecurity and unemployment, as often found during lockdown periods, are known risk factors for AF development (23, 24).

Secondly, a shift of AF patients from more specialized AF centers or cardiologic out-patient-departments to the ED could have taken place since those might have been closed during the lockdown.

### The Higher Demand of Electrical Cardioversion

Surprisingly, our data showed a significant increase of eCV rates toward the lockdown period in the intra-year analysis of 2020 (rate ratio 1.051, CI 1.008–10.96, *p* = 0.020), which cannot explained by a sudden increase in patients' age or significant dynamics in patients' gender (Table 1). This has not been reported before and stands in contrast to falling AF incidences in the same time period. Similar explanatory hypotheses apply as to the fact of AF rates not dropping as severely as in other literature: A modified lifestyle and increased stress levels during lockdown and a shift of patients from specialist centers toward the ED can be assumed. However, the increased demand of eCV parallel to dropping general AF incidences can also depict a higher necessity of rhythm control in AF patients presenting to the ED. In patients developing AF, the above mentioned factors might pose promotors of an increased chance for the current episode to be refractory to other therapeutic approaches (frequency control, electrolyte adjustment, symptomatic treatment, and even pharmacological cardioversion), therefore making eCV necessary in more cases than usual. In addition, the higher threshold of seeking medical attention might “sort out” the lesser-severe AF cases that usually also present to the ED, leaving more complex, more therapy-refractory episodes. This hypothesis could mean that—if validated in future research—special attention should be paid to those AF patients reaching medical attention regardless of a lockdown: These could be the patients needing a smooth workflow toward eCV.

### Study Limitations

The main limitation of the present analysis represents its single center setting. Our results are therefore prone to potential selection bias or further unknown factors influencing patient counts at our study center. Moreover, we do not have sufficient information about other medical centers potentially shifting AF and eCV cases to our center due to the lockdown. Also, the interesting finding of a notch in eCV numbers in March of each observed year (Figure 3) cannot be fully explained. Seasonal dynamics in AF incidences might play a role (25), but other influencing factors not identified by us can also not be ruled out. Lastly, no in-depth epidemiological patient data could be provided in order to further understand the described dynamics—this should be focus of further research.

## Conclusion

Our data of atrial fibrillation and electrical cardioversion incidences before and during a national lockdown due to COVID-19 show a trend toward decreased atrial fibrillation incidences, but a higher demand of electrical cardioversions during the lockdown period. A higher threshold of patients to seek medical attention may result in a subsequently selected group with potentially more severe clinical courses. In addition, lifestyle modifications during isolation and a higher stress level may promote atrial fibrillation episodes to be refractory to other therapeutic approaches than electrical cardioversion.

## Data Availability Statement

The raw data supporting the conclusions of this article will be made available by the authors, without undue reservation.

## Ethics Statement

The studies involving human participants were reviewed and approved by Ethics Committee of the Medical University of Vienna, Austria (EC-No. 1568/2014). Written informed consent for participation was not required for this study in accordance with the national legislation and the institutional requirements.

## Author's Note

All listed authors take responsibility for all aspects of the reliability and freedom from bias of the data presented and their discussed interpretation.

## Author Contributions

SS, JN, NS, FC, and JO collected the study data and comprised the manuscript. HD, AS, and AL comprised further manuscript versions and supervised the research process. All authors multiply revised the manuscript critically and approved of the final version.

## Conflict of Interest

The authors declare that the research was conducted in the absence of any commercial or financial relationships that could be construed as a potential conflict of interest.

## References

[1] Holt A Gislason GH Schou M Zareini B Biering-Sorensen T Phelps M. New-onset atrial fibrillation: incidence, characteristics, and related events following a national COVID-19 lockdown of 5.6 million people. Eur Heart J. (2020) 41:3072–79. 10.1093/eurheartj/ehaa494 32578859 PMC7337750

[2] Gitt AK Karcher AK Zahn R Zeymer U. Collateral damage of COVID-19-lockdown in Germany: decline of NSTE-ACS admissions. Clin Res Cardiol. (2020) 109:1585–7. 10.1007/s00392-020-01705-x 32651656 PMC7351542

[3] Metzler B Siostrzonek P Binder RK Bauer A Reinstadler SJ. Decline of acute coronary syndrome admissions in Austria since the outbreak of COVID-19: the pandemic response causes cardiac collateral damage. Eur Heart J. (2020) 41:1852–53. 10.1093/eurheartj/ehaa314 32297932 PMC7184486

[4] Austrian Ministry for Health (Bundesministerium für Soziales Gesundheit Pflege und Konsumentenschutz) Amtliches Dashboard COVID-19. (2020). Available online at: https://info.gesundheitsministerium.at/ (accessed November 07, 2020).

[5] World Health Organisation Coronavirus Disease (COVID-19) Outbreak Situation. (2020). Available online at: https://www.who.int/emergencies/diseases/novel-coronavirus-2019 (accessed November 07, 2020).

[6] Rosenbaum L. The untold toll - the pandemic's effects on patients without Covid-19. N Engl J Med. (2020) 382:2368–71. 10.1056/NEJMms2009984 32302076

[7] Nopp S Janata-Schwatczek K Prosch H Shulym I Koenigsbruegge O Pabinger I. Pulmonary embolism during the COVID-19 pandemic: Decline in diagnostic procedures and incidence at a university hospital. Res Pract Thromb Haemost. (2020) 4:835–41. 10.1002/rth2.12391 32685892 PMC7276790

[8] Kristoffersen ES Jahr SH Thommessen B Ronning OM. Effect of Covid-19 pandemic on stroke admission rates in a Norwegian population. Acta Neurol Scand. (2020) 142:632–6. 10.1111/ane.13307 32620027 PMC7361547

[9] De Ovidio F D'Ascenzo F Angelini F Bocchino PP Conrotto F Saglietto A. Reduced rate of hospital admissions for ACS during covid-19 outbreak in northern Italy. N Engl J Med. (2020) 383:88–9. 10.1056/NEJMc2009166 32343497 PMC7224608

[10] Tam C-CF Cheung K-S Lam S Wong A Yung A Sze M. Impact of coronavirus disease 2019 (COVID-19) outbreak on ST-segment-elevation myocardial infarction care in Hong Kong, China. Circ Cardiovasc Qual Outcomes. (2020) 13:e006631. 10.1161/CIRCOUTCOMES.120.006631 32182131 PMC7147280

[11] Baldi E Sechi GM Mare C Canevari F Brancaglione A Primi R. Out-of-hospital cardiac arrest during the covid-19 outbreak in Italy. N Engl J Med. (2020) 383:496–8. 10.1056/NEJMc2010418 32348640 PMC7204428

[12] Rattanawong P Shen W El Masry H Sorajja D Srivathsan K Valverde A Guidance on short-term management of atrial fibrillation in coronavirus disease J Am Heart Assoc. (2019). 2020:e017529 10.1161/JAHA.120.017529 PMC7660727 32515253

[13] Bhatla A Mayer MM Adusumalli S Hyman MC Oh E Tierney A. COVID-19 and cardiac arrhythmias. Heart Rhythm. (2020) 17:1439–44. 10.1016/j.hrthm.2020.06.016 32585191 PMC7307518

[14] Schnaubelt S Breyer M-K Siller-Matula J Domanovits H. Atrial fibrillation: a risk factor for unfavourable outcome in COVID-19? A case report. Eur. Heart J. (2020) 4:1–6. 10.1093/ehjcr/ytaa166 33089045 PMC7337643

[15] Blomström-Lundqvist C. Effects of COVID-19 lockdown strategies on management of atrial fibrillation. Eur Heart J. (2020) 41:3080–2. 10.1093/eurheartj/ehaa538 32614939 PMC7528957

[16] Kirchhof P Benussi S Kotecha D Ahlsson A Atar D Casadei B. (2016). ESC Guidelines for the management of atrial fibrillation developed in collaboration with EACTS. Eur Heart J. (2016) 37:2893–962. 10.1093/eurheartj/ehw210 27567408

[17] Wingerter R Steiger N Burrows A Estes NAM. Impact of lifestyle modification on atrial fibrillation. Am J Cardiol. (2020) 125:289–97. 10.1016/j.amjcard.2019.10.018 31761147

[18] Pellegrini M Ponzo V Rosato R Scumaci E Goitre I Benso A. Changes in weight and nutritional habits in adults with obesity during the “lockdown” period caused by the COVID-19 virus emergency. Nutrients. (2020) 12:2016. 10.3390/nu12072016 32645970 PMC7400808

[19] Mattioli AV Bonatti S Zennaro M Mattioli G. The relationship between personality, socio-economic factors, acute life stress and the development, spontaneous conversion and recurrences of acute lone atrial fibrillation. Europace. (2005) 7:211–20. 10.1016/j.eupc.2004.02.006 15878557

[20] Lima CKT Carvalho PMM Lima IAAS Nunes JVAO Saraiva JS de Souza RI. The emotional impact of Coronavirus 2019-nCoV (new Coronavirus disease). Psychiatry Res. (2020) 287:112915. 10.1016/j.psychres.2020.112915 32199182 PMC7195292

[21] Ozamiz-Etxebarria N Dosil-Santamaria M Picaza-Gorrochategui M Idoiaga-Mondragon N. Niveles de estrés, ansiedad y depresión en la primera fase del brote del COVID-19 en una muestra recogida en el norte de España. Cad Saude Publica (2020) 36:e00054020. 10.1590/0102-311x00054020 32374806

[22] Rajkumar RP. COVID-19 and mental health: a review of the existing literature. Asian J Psychiatr. (2020) 52:102066. 10.1016/j.ajp.2020.102066 32302935 PMC7151415

[23] Torén K Schiöler L Söderberg M Giang KW Rosengren A. The association between job strain and atrial fibrillation in Swedish men. Occup Environ Med. (2015) 72:177–80. 10.1136/oemed-2014-102256 25523937 PMC4345978

[24] Fransson EI Nordin M Magnusson Hanson LL Westerlund H. Job strain and atrial fibrillation - results from the Swedish longitudinal occupational survey of health and meta-analysis of three studies. Eur J Prev Cardiol. (2018) 25:1142–9. 10.1177/2047487318777387 29846118

[25] Younis A Goldenberg IL McNitt SMS Kutyifa V Polonsky B Goldenberg ID. Circadian variation and seasonal distributions of implantable defibrillator detected new onset atrial fibrillation. Pacing Clin Electrophysiol. (2020). 10.1111/pace.13995. [Epub ahead of print]. 32579238

